# Fungal association and root morphology shift stepwise during ontogenesis of orchid *Cremastra appendiculata* towards autotrophic nutrition

**DOI:** 10.1093/aobpla/plac021

**Published:** 2022-05-09

**Authors:** Franziska E Zahn, Yung-I Lee, Gerhard Gebauer

**Affiliations:** Laboratory of Isotope Biogeochemistry, Bayreuth Center of Ecology and Environmental Research (BayCEER), University of Bayreuth, Universitätsstr. 30, 95440 Bayreuth, Germany; Department of Life Science, National Taiwan University, Taipei 10617, Taiwan; Institute of Ecology and Evolutionary Biology, National Taiwan University, Taipei 10617, Taiwan; Laboratory of Isotope Biogeochemistry, Bayreuth Center of Ecology and Environmental Research (BayCEER), University of Bayreuth, Universitätsstr. 30, 95440 Bayreuth, Germany

**Keywords:** *Cremastra appendiculata*, mycoheterotrophy, mycorrhiza, ontogenesis, Orchidaceae, protocorm, rhizoctonia, saprotrophic, seedling, stable isotopes, subterranean morphology, Taiwan

## Abstract

The chlorophyllous, terrestrial orchid *Cremastra appendiculata* from East Asia is unique concerning its fungal mycorrhiza partners. The initially mycoheterotrophic protocorms exploit rather specialized non-rhizoctonia saprotrophic Psathyrellaceae. Adult individuals of this orchid species are either linked to Psathyrellaceae being partially mycoheterotrophic or form mycorrhiza with fungi of the ubiquitous saprotrophic rhizoctonia group. This study provides new insights on nutrition mode, subterranean morphology and fungal partners across different life stages of *C. appendiculata*. We compared different development stages of *C. appendiculata* to surrounding autotrophic reference plants based on multi-element natural abundance stable isotope analyses (δ^13^C, δ^15^N, δ^2^H, δ^18^O) and total N concentrations. Site- and sampling-time-independent enrichment factors of stable isotopes were used to reveal trophic strategies. We determined mycorrhizal fungi of *C. appendiculata* protocorm, seedling and adult samples using high-throughput DNA sequencing. We identified saprotrophic non-rhizoctonia Psathyrellaceae as dominant mycorrhizal fungi in protocorm and seedling rhizomes. In contrast, the roots of seedlings and mature *C. appendiculata* were mainly colonized with fungi belonging to the polyphyletic assembly of rhizoctonia (*Ceratobasidium*, *Thanatephorus* and Serendipitaceae). Mature *C. appendiculata* did not differ in isotopic signature from autotrophic reference plants suggesting a fully autotrophic nutrition mode. Characteristic of orchid specimens entirely relying on fungal nutrition, *C. appendiculata* protocorms were enriched in ^15^N, ^13^C and ^2^H compared to reference plants. Seedlings showed an intermediate isotopic signature, underpinning the differences in the fungal community depending on their subterranean morphology. In contrast to the suggestion that *C. appendiculata* is a partially mycoheterotrophic orchid species, we provide novel evidence that mature *C. appendiculata* with rhizoctonia mycobionts can be entirely autotrophic. Besides an environmentally driven variability among populations, we suggest high within-individual flexibility in nutrition and mycobionts of *C. appendiculata*, which is subject to the ontogenetic development stage.

## Teaser

The green orchid *Cremastra appendiculata*from East Asia is unique, because it changes fungal mycorrhiza partners during development. In the early seedling stage this orchid exploits wood- or litter-decomposing fungi, while adults either continue being linked to these wood- or litter-decomposers or switch towards ubiquitous saprotrophic fungi of the rhizoctonia group. This study provides new insights on changes in subterranean morphology and nutrition mode accompanying this fungal partner switch. It turned out that only rhizomes were colonized by the wood- or litter-decomposing fungi while roots of adults were colonized by rhizoctonia. While early seedlings fully relied on fungi as nutrient source, adults without rhizomes were fully autotrophic.

## Introduction

The Orchidaceae are one of the largest and most diverse plant families on Earth, containing an estimate of roughly 28 500 species (WCSP 2021). It is particularly fascinating that orchids exhibit different trophic strategies at both phylogenetic and ontogenetic level (Dearnaley *et al.* 2012; Field *et al.* 2017).

In this sense, probably all representatives within the Orchidaceae rely on carbon (C) gained through the interaction with associated fungi during their early below-ground development stage (Rasmussen 1995; Leake 2004; Rasmussen *et al.* 2015). This initially mycoheterotrophic nutrition mode may be linked to the ‘dust seeds’ of orchids, which contain an embryo but lack an endosperm, resulting in very limited carbohydrate reserves (Leake 1994; Arditti and Ghani 2000; Eriksson and Kainulainen 2011). Even after ‘symbiotic germination’, orchids remain mycoheterotrophic throughout their non-photosynthetic protocorm phase (Dearnaley *et al.* 2012). The dependency of orchids on fungal carbon usually decreases towards adulthood (Rasmussen *et al.* 2015). However, over 200 orchid species from several genera remain achlorophyllous and thus fully mycoheterotrophic for their entire life span.

Achlorophyllous, fully mycoheterotrophic orchid specimens tend to exploit ectomycorrhizal networks (ECM) in temperate latitudes, while in the (sub)tropics additionally non-rhizoctonia litter- and wood-decaying saprotrophic fungi (SAP) seem to be important associating fungi (Martos *et al.* 2009; Ogura-Tsujita *et al.* 2009, 2018; Lee *et al.* 2015).

Nonetheless, most orchids develop chlorophyll and the ability to photosynthesize, becoming either autotrophic or, when getting additional carbon from fungi, partially mycoheterotrophic (Gebauer and Meyer 2003; Merckx *et al.* 2009). Amongst autotrophic or partially mycoheterotrophic orchids, saprotrophic rhizoctonia and to a lower extent fungi simultaneously forming ectomycorrhizas with forest trees are fungal partners (Bidartondo *et al.* 2004). Mycorrhizal associations with the polyphyletic fungal assembly of rhizoctonia, comprising saprotrophic fungi belonging to the Tulasnellaceae, Ceratobasidiaceae and Serendipitaceae within the Basidiomycetes, are thus the most common group involved in orchid mycorrhiza and are unique to Orchidaceae (Rasmussen 2002; Taylor *et al.* 2002; Dearnaley *et al.* 2012). Orchid mycorrhizal interactions likely emerged by the evolutionary recruitment of endophytes that became mycorrhizal (Selosse *et al.* 2021). Generally, shifts of fungal partners among Orchidaceae often co-occur with switches in trophic modes (Wang *et al.* 2021).

It is understood that conclusions on the evolution from autotrophy to mycoheterotrophy and the relevance of changing mycorrhizal fungi can be drawn by studying leafy and leafless plant relatives as well as ontogenetic changes in fungal mycorrhiza associations within one plant species (Ogura-Tsujita *et al.* 2021). Covering two achlorophyllous, leafless and three chlorophyllous, leafy species, the orchid genus *Cremastra* from East Asia is such a prime example of SAP–mycoheterotrophic plant lineages (Ogura-Tsujita *et al.* 2021; Suetsugu 2021).

*Coprinellus* spp., rather specialized wood-/litter-decaying saprotrophic fungi belonging to the Psathyrellaceae, have been identified as fungal hosts of the fully mycoheterotrophic, leafless orchid *Cremastra aphylla* (Yagame *et al.* 2018; Suetsugu *et al.* 2021a). Consistently, *Coprinellus* spp. are known to induce seed germination in the protocorm stage of chlorophyllous *Cremastra appendiculata* (Yagame *et al.* 2013). Chlorophyllous *C. appendiculata* with relatively broad green leaves is usually found in light-limited and humid forest ground sites in the (sub)tropics and was therefore considered to be putatively partially mycoheterotrophic as an adult. Recently, mature *C. appendiculata* var. *variabilis* individuals sampled in a cool-temperate forest in Japan were recognized to obtain approximately 83.4 ± 0.9 % of their total C demand from wood-decaying Psathyrellaceae (*Psathyrella* or *Coprinellus*) (Suetsugu *et al.* 2021a). Though confirming partial mycoheterotrophy for several individuals with Psathyrellaceae as fungal partners, Yagame *et al.* (2021) identified rhizoctonia (Tulasnellaceae, Ceratobasidiaceae, Serendipitaceae) as the main mycobionts of *C. appendiculata* var. *variabilis*.

Strikingly, Psathyrellaceae fungi were exclusively detected in mature *C. appendiculata* var. *variabilis* individuals with coralloid rhizomes (Suetsugu *et al.* 2021a; Yagame *et al.* 2021), a morphological root structure typical for mycoheterotrophic plants (Burgeff 1932; Leake 1994), while rhizoctonia were mainly found in individuals without coralloid rhizomes (Yagame *et al.* 2021). Therefore, Yagame *et al.* (2021) suggested an environmentally driven link between fungal partners and subterranean morphology.

**Figure 1.**
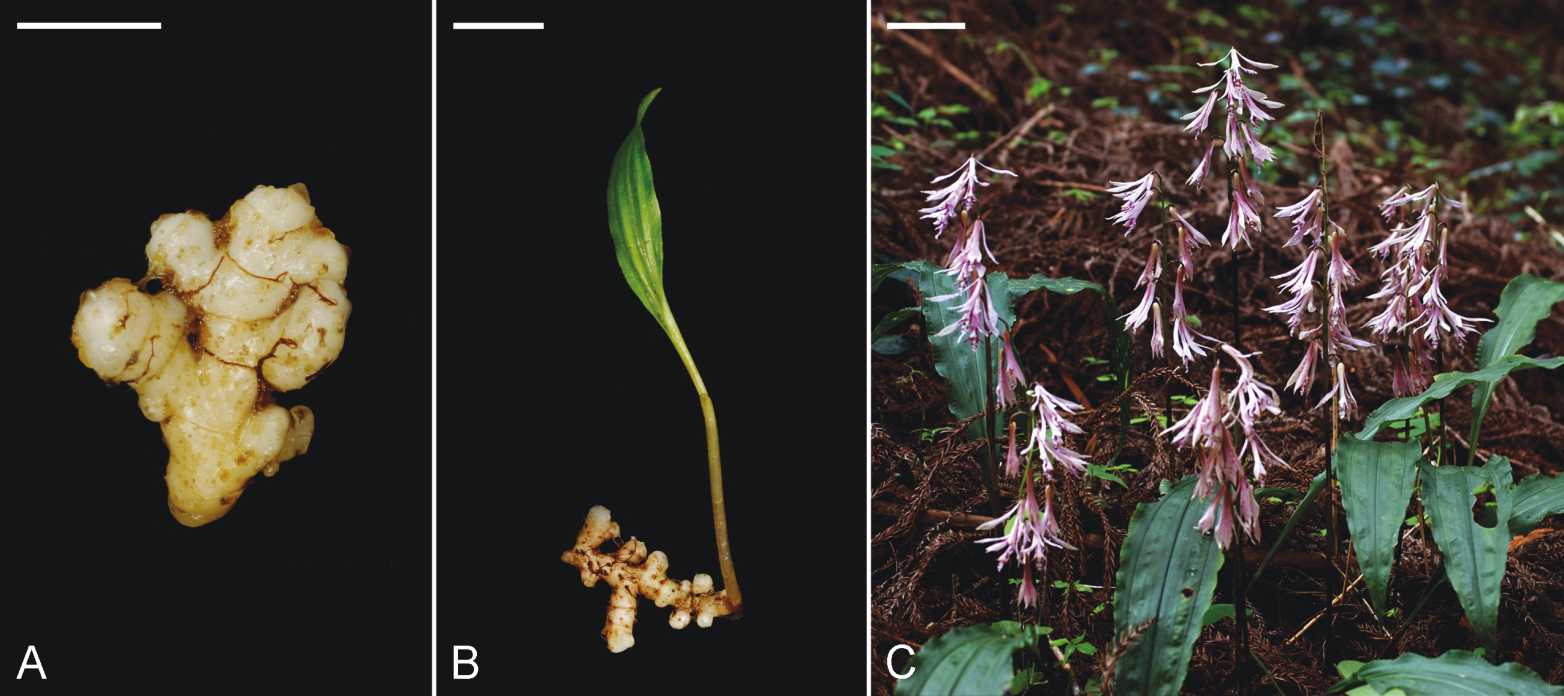
Development stages of *Cremastra appendiculata*. (A) Protocorm. (B) Seedling with very early leaf. (C) Flowering mature individuals; scale: A = 5 mm; B = 5 cm; C = 5 cm.

This study provides new insights on nutrition mode, underground morphology and fungal partners across three different life stages (protocorms, seedlings, adults) of *C. appendiculata*. We used multi-element natural abundance stable isotope analysis together with high-throughput DNA sequencing to investigate field-collected protocorms, seedlings and adults of a *C. appendiculata* population (Fig. 1) in a Fagaceae forest habitat in the Taiwanese mountains.

Analysing δ^2^H and δ^18^O additionally to δ^13^C and δ^15^N enabled us to resolve presumed ‘cryptic partial mycoheterotrophy’ (*sensu*Hynson *et al.* 2013) of mature *C. appendiculata* being mycorrhizal with rhizoctonia. This approach provides specific evidence whether mature *C. appendiculata* adults with rhizoctonia fungal partners continue to gain some organic matter from a fungal source or whether they are truly autotrophic (Gebauer *et al.* 2016; Schiebold *et al.* 2018). We examined whether a change of fungal partners along three different ontogenetic development stages of *C. appendiculata* is accompanied by an alteration in subterranean root morphology and the mode of nutrition. We assume a change in carbon acquisition to be visualized by gradual changes in stable isotope natural abundance.

## Materials and Methods

### Sampling sites and experimental design

Sampling of *C. appendiculata* var. *variabilis* at three different development stages and respective autotrophic reference plants (listed in **Supporting Information—Tables S3****and****S4**) took place in a Fagaceae forest in subtropical Taiwan on the western slope of the Hehuan Mountains at an elevation of 2000 m a.s.l. (24°05ʹ01.4″N, 121°10ʹ10.2″E).

An *in situ* sowing experiment yielded protocorm samples of *C. appendiculata* (Fig. 1A). For this purpose, seed packages of *C. appendiculata* were buried into the soil and covered by litter near the adult at the sampling site. As seeds matured in late October, seeds were collected from 15 capsules, pooled after harvesting and directly put into seed packages. Approximately 300 seeds were placed within a seed package. Seed packages were made of mesh permeable for fungal hyphae but not for plant roots (Rasmussen and Whigham 1998). About 100 seed packages were buried in 2012, 2013, 2014 and 2015 at five plots. As the seed packages were retrieved, they were smoothly rinsed with tap water in the laboratory, then opened and carefully checked under a stereomicroscope for examining germination.

The sampling design for stable isotope analysis followed the approach of Gebauer and Meyer (2003), consisting of 1 m^2^ plots with the target orchid species and four to five autotrophic reference plant species. This sampling scheme enables sufficient replicates of the target orchid. It ensures almost identical microclimatic conditions, soil properties and light availability of the target orchids and the respective reference plants to avoid bias on ^2^H, ^13^C and ^15^N relative abundance due to microsite differences (Dawson *et al.* 2002). Autotrophic non-orchid reference plants were chosen according to the criteria discussed by Gebauer and Meyer (2003) and Hynson *et al.* (2013). We strived to cover a broad spectrum of growth habits, life forms and taxonomy. We took leaf samples from five flowering mature *C. appendiculata* individuals in 2 June 2011 (Fig. 1C) and from three *C. appendiculata* seedlings with very early green leaves in 19 July 2015 (Fig. 1B), respectively. The seven analysed protocorms originate from seed packages buried *in situ* for at least 3 years (harvested in October of 2015 and 2019).

For the high-throughput sequencing experiment, samples were taken from the above-mentioned plots on 19 July 2015, and we differentiated between root morphology types due to variation in the investigated population depending on the development stage. Note that seedlings with very young leaves had both coralloid rhizomes and roots attached, but as seedlings approached maturity, they became detached from rhizomes. Two to three protocorms were collected from each plot (eight protocorms in total from three plots). The protocorms from seed packets in each plot were pooled because of the small amount of tissue. Only three plots with the seedling stage were available, and one coralloid rhizome and one root were collected from each individual in each plot (three coralloid rhizomes and three roots in total from three plots). The colonized samples from coralloid rhizomes were pooled to create a homogeneous mycorrhizal coralloid rhizome sample. The distal 3-cm portions of colonized roots were sectioned into 3-mm fragments, which were then combined to a homogeneous mycorrhizal root sample. For the adult plant sample without coralloid rhizomes, three roots of each individual were collected in each plot (15 roots from five individuals in total from five plots), colonized roots were sectioned and pooled.

### Molecular identification of mycorrhizal fungi

#### High-throughput sequencing.

The surface of roots and protocorms was washed in tap water and subsequently sterilized with a 1 % sodium hypochlorite solution for 60 s, followed by three 60-s rinse steps in sterile distilled water and microscopically checked for mycorrhizal colonization. Afterwards, DNA was extracted from 0.05–0.1 g mycorrhizal samples using the Plant Genomic DNA Purification Kit as described by the manufacturer (GMbiolab Co. Ltd, Taichung, Taiwan).

The internal transcribed spacer 1 (ITS1) region of the nuclear ribosomal RNA genes was amplified using the primer pair ITS1F and ITS2R (**see****Supporting Information—Table S2**; Adams *et al.* 2013). PCR was carried out in 20 μL reactions containing 10 ng of genomic DNA, 0.8 μL of each primer (5 μM), 2 μL of 2.5 mM dNTPs, 4 μL of 5× TransStart® FastPfu Buffer (TransGen Biotech Co., Ltd, Beijing, China) and 0.4 μL of TransStart® FastPfu Polymerase. The parameters of reactions consisted of an initial denaturation at 95 °C for 3 min, then 35 cycles of 95 °C for 30 s, and 55 °C for 30 s and a final extension of 72 °C for 45 s. PCR products were separated by gel electrophoresis, and amplicons within the appropriate size range were cut and purified using the AxyPrep DNA Gel Extraction Kit (GMbiolab Co. Ltd, Taichung, Taiwan) and quantified using QuantiFluor™ Fluorometer (Promega Corporation, Madison, WI, USA). Samples were then pooled in equimolar concentrations and paired-end sequenced (2 × 250 bp) on an Illumina Miseq. Raw fastq files were demultiplexed and quality-filtered using QIIME (version 1.17) with the following criteria. First, 300-bp reads were truncated at any site receiving an average quality score < 20 over a 50-bp sliding window, discarding the truncated reads shorter than 50 bp. Second, reads with one or two nucleotide mismatches in primer matching measurements and reads containing ambiguous characters were removed. Third, only sequences that had an overlap longer than 10 bp were assembled according to their overlap sequence. Reads that could not be assembled were discarded.

#### Bioinformatics.

Sequences obtained from the Illumina Miseq run were clustered into operational taxonomic units (OTUs) using the UPARSE algorithm implemented in USEARCH version 7 (Edgar 2013). Operational taxonomic units were clustered with 97 % similarity cut-off using UPARSE (version 7.1; http://drive5.com/uparse/, and chimaeras were identified and removed using the UNITE UCHIME reference data set. The taxonomy of each ITS1 region was analysed by RDP Classifier (http://rdp.cme.msu.edu/) against the UNITE fungal ITS database using a confidence threshold of 70 %. Remaining OTUs were assigned taxonomic identities based on the top 10 BLAST (megablast algorithm) (Altschul *et al.* 1990) results of the OTU representative sequences (selected by UPARSE) using the GenBank nucleotide (nt) database (Benson *et al.* 2012), including uncultured/environmental entries. Operational taxonomic units identified by BLAST were then manually screened for potential orchid-associating mycorrhizal families following a stepwise process. Firstly, OTUs represented by short sequences (<150 bp) or having a low sequence similarity (<90 %) with fungal species across their sequence length were removed. Secondly, only OTUs found on at least one orchid root sample in this study were retained.

#### Phylogenetic analysis.

To test phylogenetic hypotheses, we generated complete ITS sequences from the DNA extractions of mycorrhizal samples. The ITS region of the fungal nuclear ribosomal RNA gene was amplified with the primer combinations ITS1F/ITS4 (Gardes and Bruns 1993) or ITS1-OF/ITS4-OF (Taylor and McCormick 2008). PCR amplification and sequencing were carried out as described by Yagame *et al.* (2016). PCR products that were difficult to sequence directly were cloned using the pGEM-T Vector System II (Promega, Madison, WI, USA). For phylogenetic analysis, ITS sequences of Psathyrellaceae from GenBank were added to the analysis by referring to Yagame *et al.* (2013) and Suetsugu *et al.* (2021a, 2021b), and the sequence of *Parasola leiocephala* was used as the outgroup taxon. ITS sequences of Ceratobasidiaceae from GenBank were added to the analysis by referring to Suetsugu and Matsubayashi (2020), and the sequence of *Botryobasidium subcoronatum* was used as the outgroup taxon. ITS sequences of Sebacinales from GenBank were added to the analysis by referring to Yagame *et al.* (2016), and the sequence of *Auricularia auricula-judae* was used as the outgroup taxon. The sequence data were aligned using CLUSTALX (Thompson *et al.* 1997), followed by manual adjustment. Phylogenetic analyses were conducted by a model-based Bayesian method using MrBayes 3.2.1 (Ronquist and Huelsenbeck 2003). The ‘best-fit’ model of evolution was selected under the Akaike information criterion test (Akaike 1974) as implemented in MrModeltest 2.2 (Nylander 2004), and the general time-reversal plus invariant rates and a gamma distribution (GTR + I + Γ) was selected for the analyses. Two separate runs of four Monte Carlo Markov chains (Yang and Rannala 1997) were performed for 10 000 000 generations until the mean deviation of split frequency dropped below 0.01, and a tree was sampled every 1000th generation. Trees from the first 25 % of generations were discarded using the ‘burn-in’ command. The remaining trees were used to calculate a 50 % majority-rule consensus topology and determine posterior probabilities for individual branches.

### Multi-element stable isotope analyses

For multi-element stable isotope analysis, sample preparation was according to **Supporting Information—Table S1**. Relative natural abundance analysis of carbon (^13^C/^12^C) and nitrogen (^15^N/^14^N) isotopes was determined simultaneously using an EA-IRMS coupling according to Bidartondo *et al.* (2004) and **Supporting Information—Table S1**. Relative natural abundances of hydrogen (^2^H/^1^H) and oxygen isotopes (^18^O/^16^O) of each plant sample were measured using a TC-IRMS coupling as described in **Supporting Information—Table S1**. The oxygen isotope abundances were measured to assess a potential transpiration effect on the relative enrichment in ^13^C and ^2^H caused by differences in stomatal regulation and transpiration between orchids and the non-orchid reference plants. Transpiration affects ^13^C, ^2^H and ^18^O isotope abundance in plant tissues simultaneously (da Silveira Lobo Sternberg 1988; Ziegler 1989; Flanagan *et al.* 1991; Cernusak *et al.* 2004). A transpiration effect can only be precluded if the ^18^O isotope abundance of the target species is similarly distributed as the ^18^O isotope signature of the non-orchid reference plants (Gebauer *et al.* 2016). Measured relative isotope abundances are denoted as δ values that were calculated according to the following equation: δ^13^C, δ^15^N, δ^2^H or δ^18^O = (*R*_sample_/*R*_standard_ − 1) × 1000 ‰, where *R*_sample_ and *R*_standard_ are the ratios of heavy to the light isotope of the samples and the respective standard. The reference sampling system allowed achieving year- and site-independent and thus comparable stable isotope data of the potentially mycoheterotrophic plants by calculating enrichment factors (ε) from the measured δ values as follows: ε = δS – δREF, where δS is a single δ^13^C, δ^15^N, δ^2^H or δ^18^O value of an orchid individual or an autotrophic reference plant and δREF is the mean value of all autotrophic reference plants by plot (Preiss and Gebauer 2008). The ε approach was essential because sampling of different development stages within 1 year was not feasible. All single values and mean values ± standard deviations of δ^13^C, δ^15^N, δ^2^H and δ^18^O, of enrichment factors ε^13^C, ε^15^N, ε^2^H and ε^18^O, and of total N concentrations of the investigated orchid species and their autotrophic references are available in **Supporting Information—Tables S2****and****S3**.

We tested for pairwise differences in the isotopic enrichment factors ε^13^C, ε^15^N, ε^2^H and ε^18^O between orchid specimens and all autotrophic reference plants applying non-parametric statistical tests because Shapiro–Wilk tests revealed non-normality. Autotrophic reference plants and *C. appendiculata* protocorm samples from different years, respectively, were treated as one group each due to insignificant differences within groups. After a significant Kruskal–Wallis *H*-test, pairwise differences between mature *C. appendiculata*, seedlings, protocorms and autotrophic references were assessed performing a *post hoc* Mann–Whitney *U*-test. Because of the relatively small sample size and few groups, we assumed a low risk of false positives and did not correct for multiple testing. We compared N concentrations of different groups using the Student’s *t*-test since Shapiro–Wilk test and Bartlett’s test confirmed normal distribution and homogeneity of variance, respectively. Statistical analyses and plotting were carried out in R Version 4.0.3 (R Core Team 2020) with a significance level of α = 0.05.

## Results

### Molecular identification of mycorrhizal fungi and germination mycobionts

The quality-filtered and normalized Miseq data set contained samples from protocorms, coralloid rhizomes of seedlings, roots of seedlings and roots of adult plants. The protocorm sample comprised 9 OTUs (41 093 sequences), the coralloid rhizomes of the seedling sample comprised 9 OTUs (41 078 sequences), the roots of the seedling sample comprised 35 OTUs (41 005 sequences) and the roots of the adult plant sample comprised 21 OTUs (41 127 sequences) (excluding OTUs with <200 total sequences; **see****Supporting Information—Table S2**).The rarefaction curve analysis indicated that the numbers of OTUs were close to saturation after 20000 sequence counts **[see****Supporting Information—Fig. S1****]**.

**Figure 2.**
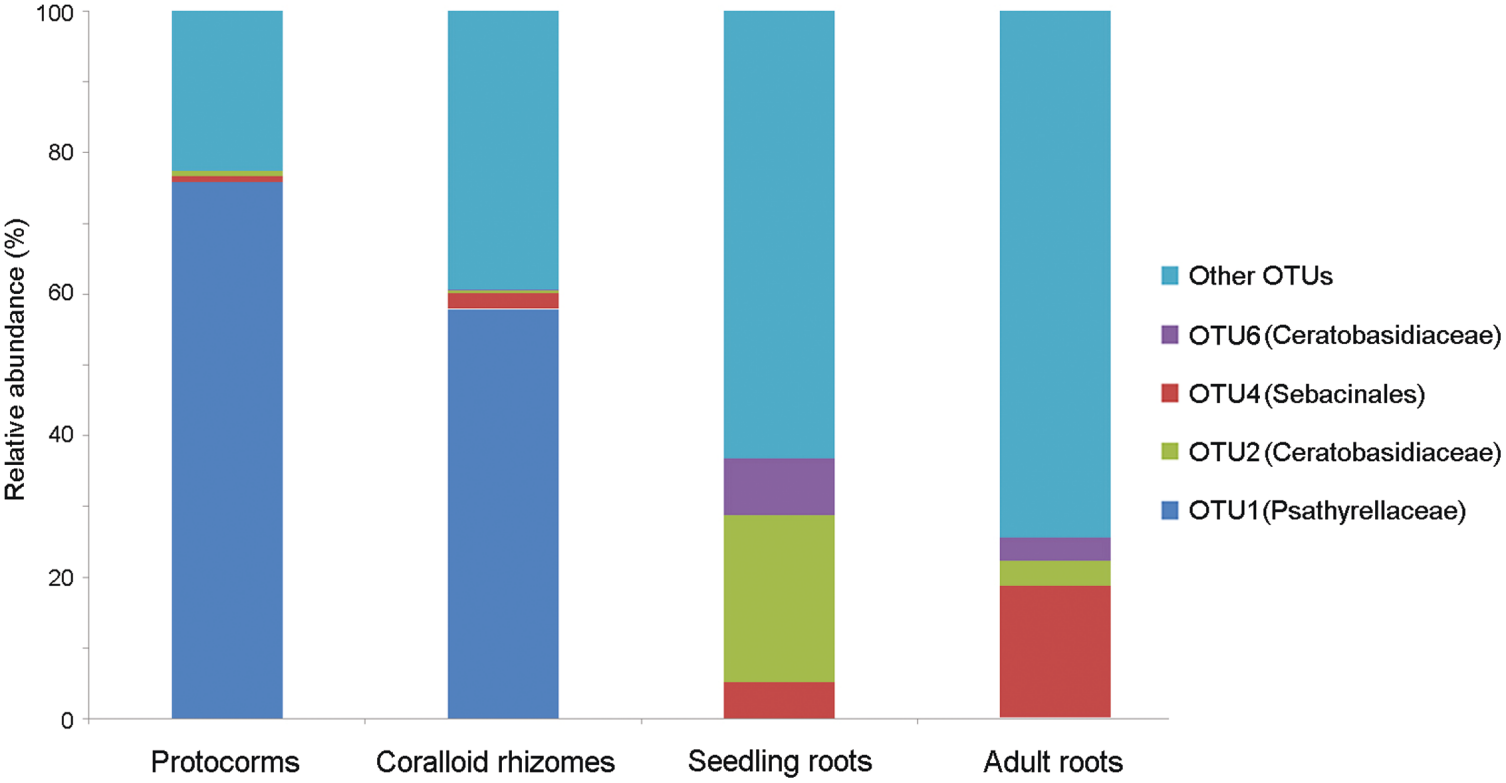
The proportion of putative orchid mycorrhizal fungi, including OTU1 (Psathyrellaceae), OTU2 (Ceratobasidiaceae), OTU4 (Sebacinales) and OTU6 (Ceratobasidiaceae) detected in protocorms, seedling rhizomes, seedling roots and adult roots of *Cremastra appendiculata* in central Taiwan. Most of other OTUs are Ascomycota, not related to the fungal taxa in orchid mycorrhizal association in the published database.

In the protocorm sample, the OTU with the highest number of reads belonged to Psathyrellaceae (OTU1—31 108 sequences, 75.7 %), while the typical orchid mycorrhizal families, Ceratobasidiaceae (OTU2—285 sequences, 0.69 %) and Sebacinales (OTU4—370 sequences, 0.9 %) were less abundant. In the coralloid rhizome sample of seedlings, the OTU with the highest number of reads also belonged to Psathyrellaceae (OTU1—23 767 sequences, 57.86 %), whereas the typical orchid mycorrhizal families, Ceratobasidiaceae (OTU2—188 sequences, 0.46 %), Ceratobasidiaceae (OTU6—2 sequences, 0.005 %), Sebacinales (OTU4—914 sequences, 2.23 %) had lower proportions (Fig. 2). On the contrary, in the root samples of seedlings and adult plants, the typical orchid mycorrhizal fungi, i.e. Ceratobasidiaceae and Sebacinales, were the most dominant fungal partners (Fig. 2). In the root sample of seedlings, the proportions of two OTUs of Ceratobasidiaceae were higher than in adult root samples, i.e. OTU2 (9708 sequences, 23.68 %) and OTU6 (3306 sequences, 8.06 %). The proportion of Sebacinales also increased from seedling to adult root samples (OTU4—2039 sequences, 4.97 %). In the root sample of adult plants, the OTU with the highest number of reads also belonged to Sebacinales (OTU4—7592 sequences, 18.45 %), followed by two OTUs of Ceratobasidiaceae, i.e. OTU2 (1439 sequences, 3.51 %) and OTU6 (1360 sequences, 3.31 %).

Phylogenetic analyses revealed that the fungal sequence from protocorm and rhizome samples demonstrated a high DNA sequence homology with the fungal genus *Coprinellus* (Psathyrellaceae) **[see****Supporting Information—Fig. S2****]**. In root samples, two fungal sequences were related to the genera *Ceratobasidium* and *Thanatephorus* but not grouped with the ECM-forming clade of Ceratobasidiaceae **[see****Supporting Information—Fig. S4****]**, suggesting the role of saprotrophic fungi. Besides, another fungal sequence from root samples belonged to Serendipitaceae **[see****Supporting Information—Fig. S3****]**.

### Stable isotope natural abundances and total N concentrations

Significant patterns emerged when examining stable isotope natural abundances and N concentrations of investigated orchid specimens and autotrophic reference plants (Table 1).

**Table 1.** Pairwise comparisons of ε^13^C, ε^15^N, ε^2^H and ε^18^O between the three development stages of *Cremastra appendiculata* (protocorms, seedlings, adults) and autotrophic reference plants using the Mann–Whitney *U*-test after a significant Kruskal–Wallis *H*-test (ε^13^C: *H* = 22.821, df = 3, *P* < 0.001; ε^15^N: *H* = 22.354, df = 3, *P* < 0.001; ε^2^H: *H* = 12.802, df = 2, *P* = 0.002; ε^18^O: *H* = 9.6663, df = 2, *P* = 0.008).

	ε^15^N		ε^13^C		ε^2^H		ε^18^O	
	*U*	*P*	*U*	*P*	*U*	*P*	*U*	*P*
Protocorm vs. reference	281	**<0.001**	281	**<0.001**	281	**<0.001**	281	**<0.001**
Seedling vs. reference	108	**0.008**	82	0.182	NA	NA	NA	NA
Adult vs. reference	119	0.313	132	0.130	74	0.552	33	0.108
Protocorm vs. seedling	6	0.279	24	**0.012**	NA	NA	NA	NA
Protocorm vs. adult	39	**0.003**	40	**0.002**	25	**0.008**	0	**0.008**
Seedling vs. adult	15	**0.036**	10	0.571	NA	NA	NA	NA

Significances are highlighted in bold.

**Figure 3.**
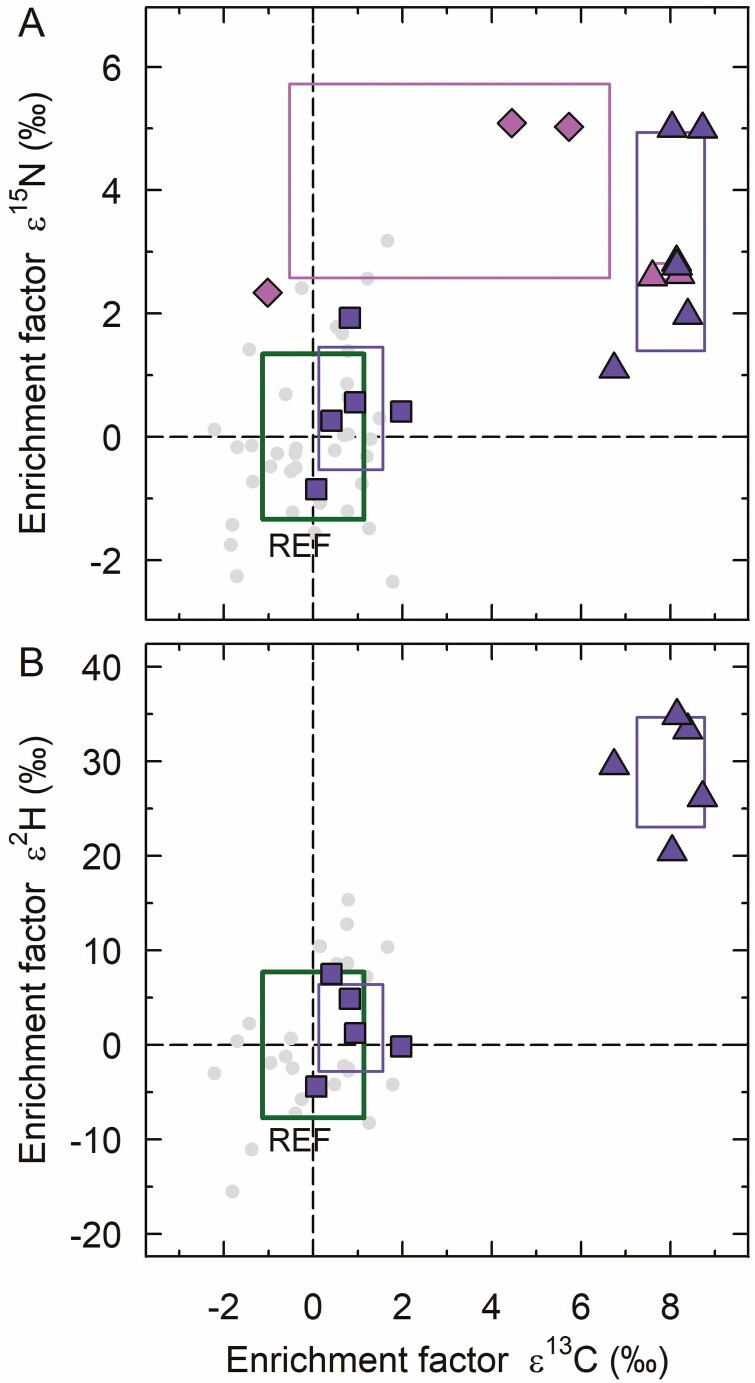
Carbon and nitrogen (A) and carbon and hydrogen (B) stable isotope enrichment factors ε of *Cremastra appendiculata* protocorms (triangle), seedlings (diamonds) and adults (squares), and respective reference plants (REF, dots). Frames represent the standard deviation from the mean enrichment factors ε of each group, while each symbol denotes a single plant individual. Identical colours represent same sampling plot scheme (dark purple: 2011/19, light purple: 2015). The green frame represents the standard deviation of autotrophic reference plants around a mean enrichment factor of zero, by definition. Data on hydrogen stable isotope enrichment factors ε of seedlings and of protocorms from 2015 are not available due to material limitation of these samples. For colour figure refer to online version.

Mycoheterotrophic protocorms of *C. appendiculata* sampled in 2019 and 2015 exhibited similar enrichment factors on average with ε^15^N of 3.16 ± 1.77 ‰ and 2.68 ± 0.12 ‰, respectively, and ε^13^C of 8.01 ± 0.76 ‰ and 7.98 ± 0.34 ‰, respectively (Fig. 3A). Protocorm stage of *C. appendiculata* showed significant enrichment in ^15^N and ^13^C compared to autotrophic reference plants (*U* = 281, *P* < 0.001 and *U* = 298, *P* < 0.001). While protocorms of *C. appendiculata* from 2019 were on average 28.83 ± 5.80 ‰ significantly enriched in ^2^H (*U* = 125, *P* < 0.001), they were depleted in ^18^O relative to the reference plants (*U* = 15, *P* = 0.006) (Fig. 3B; Table 1).

Conversely, none of the stable isotope signatures of mature *C. appendiculata* differed statistically from autotrophic reference plants (Table 1; Fig. 3A and B).

*Cremastra appendiculata* seedlings had on average an ε^15^N of 4.15 ± 1.57 ‰, being significantly enriched relative to autotrophic reference plants (*U* = 108, *P* = 0.008). Therefore, ^15^N enrichment of seedlings was in a similar range as ^15^N enrichment of protocorms (Fig. 3A). Seedling samples were non-significantly enriched in ^13^C relative to reference plants (*U* = 82, *P* = 0.182), displaying an intermediate position between protocorms and mature *C. appendiculata* (Fig. 3A). Enrichment factor ε^13^C of seedlings showed a rather considerable variation (3.06 ± 3.59 ‰) and was only statistically distinct from protocorms (*U* = 24, *P* = 0.012).

**Figure 4.**
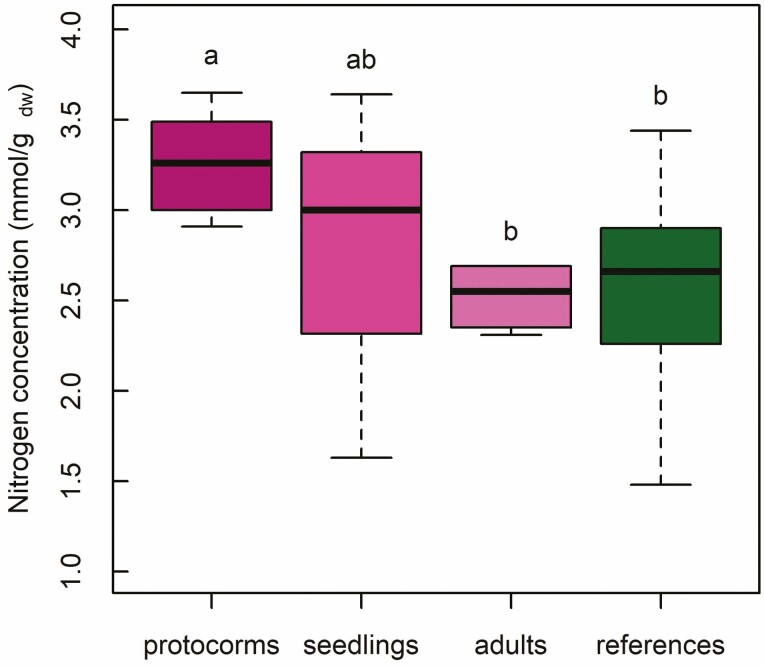
Nitrogen concentration (total N) for *Cremastra appendiculata* protocorms, seedlings, adults and reference plants. The box spans the first and third quartile, while the horizontal line in the box represents the median; whiskers extend to 1.5 * interquartile range. Different letters indicate statistically significant differences (Student’s *t*-test) between groups.

Total N concentration was on average highest in *C. appendiculata* protocorms (3.26 ± 0.28 mmol per g dry weight), followed by leaf N concentrations of seedlings with the largest variation (2.76 ± 1.23 mmol per g dry weight). Total N concentration of adult *C. appendiculata* (2.52 ± 0.18 mmol per g dry weight) and autotrophic reference plants (2.53 ± 0.49 mmol per g dry weight) were on average similar. They were statistically distinct from total N concentrations in protocorms (Fig. 4; **see****Supporting Information—Table S3**).

## Discussion

In this study, we assessed for the first time fungal associates, subterranean morphology and nutrition mode of three different ontogenetic development stages of *C. appendiculata* within one population: protocorms, seedlings as an intermediate stage and adults.

### Initially mycoheterotrophic protocorm stage in the natural environment

Saprotrophic *Coprinellus* spp. seem to be substantial mycobionts involved in the mycoheterotrophic nutrition mode within the *Cremastra* genus. They are fungal hosts of fully mycoheterotrophic orchid *C. aphylla* (Yagame *et al.* 2018) and partially mycoheterotrophic mature *C. appendiculata* (Suetsugu *et al.* 2021a; Yagame *et al.* 2021). Identifying wood-/litter-decaying *Coprinellus* fungi as dominant mycobionts in *C. appendiculata* protocorm–rhizomes directly from an *in situ* experimental set-up is new to science but in accordance with earlier findings from symbiotic laboratory cultivation (Yagame *et al.* 2013).

Characteristic for mycoheterotrophic orchid specimens, which gain carbohydrates additionally to other nutrients from a fungal partner, *C. appendiculata* protocorms showed enrichment in ^15^N, ^13^C and ^2^H and elevated leaf N concentrations. Here we present the first stable isotope patterns of protocorms related to non-rhizoctonia saprotrophic fungal partners, which are distinct from those of protocorms and fully mycoheterotrophic (FMH) adult orchids with rhizoctonia and ECM fungal partners (Table 2). ^15^N and ^13^C signatures of *C. appendiculata* protocorms were rather within the range of FMH orchids with non-rhizoctonia SAP fungal partners; however, the substrate of the fungal partner (wood or litter) cannot clearly be distinguished by stable isotopes pattern hitherto (Table 2). Future radiocarbon measurements may reveal whether *C. appendiculata* protocorms gain carbon indirectly from litter or dead wood (Hatté *et al.* 2020; Suetsugu *et al.* 2020), with the latter being likely because dead wood was already shown as the source for *Psathyrella* and *Coprinellus* fungal partners of mature, partially mycoheterotrophic *C. appendiculata* and fully mycoheterotrophic *C. aphylla* from Japan (Suetsugu *et al.* 2021a)*.*

**Table 2.** Mean enrichment factors ε^15^N, ε^13^C and ε^2^H (bold) *±* SD (italic) of adult leaves and protocorms of here investigated *Cremastra appendiculata* and Orchidaceae specimens extracted from published literature until November 2021 for comparison. Comparative values of orchid specimens are grouped by their type of fungal partner: Association with saprotrophic wood- or litter-decomposing fungi (SAP wood/litter), association with ectomycorrhizal fungi of trees (ECM), association with rhizoctonia. FMH indicate fully mycoheterotrophic, achlorophyllous orchid species. IMH indicate initially mycoheterotrophic protocorms.

		*n*_s_	*n*_spp._	ε^15^N	ε^13^C	*n*_s_	*n*_spp._	ε^2^H
*C. appendiculata* protocorm		*8*	*1*	**2.98** ± *1.36*	**8.00** *± 0.60*	5	*1*	**28.83** *± 5.8*
*C. appendiculata* adult		*5*	*1*	**0.46** *± 0.99*	**0.84** *± 0.99*	5	*1*	**1.80** *± 4.59*
SAP wood	FMH adult	*43*	*9*	**4.29** *± 2.09*	**10.98** *±* 2.30	*NA*	*NA*	*NA*
SAP litter	FMH adult	*15*	*3*	**4.99** *± 0.53*	**8.24** *±* 0.49	*NA*	*NA*	*NA*
ECM	IMH protocorm	*46*	*2*	**7.48** *± 2.26*	**7.55** *±* 0.62	*NA*	*NA*	*NA*
	FMH adult	*163*	*15*	**11.78** *± 3.22*	**7.82** *±* 1.57	*10*	*1*	**57.30** *± 13.83*
Rhizoctonia	IMH protocorm	*49*	*6*	**5.77** *± 2.73*	**6.12** *±* 2.70	*5*	*1*	**95.78** *± 6.22*
	FMH adult	*9*	*2*	**2.20** *± 2.46*	**9.27** *±* 0.75	*NA*	*NA*	*NA*
	Green adult	*674*	*65*	**2.94** *± 2.30*	**−0.48** *±* 1.90	136	*17*	**18.71** *± 12.95*

Data source is according to Schweiger (2018) and extended by data from Suetsugu *et al.* (2019), Suetsugu *et al.* (2020), Jacquemyn *et al.* (2021), Suetsugu and Matsubayashi (2021b), Suetsugu *et al.* (2021a) and Suetsugu *et al.* (2021b).

### Seedlings—intermediate stage ‘mirroring the variability of adults’

During seedling growth of orchids, there can be narrow checkpoints for mycorrhizal range relative to the more promiscuous germination and mature stages, e.g. for *Epipactis helleborine* (Bidartondo and Read 2008). However, partial replacement of mycobionts in the seedling stage likely reduces the risk of failing to find new mycobionts during ontogenesis (McCormick *et al.* 2006) and is the most frequent scenario of temporal turnover in orchid–mycorrhizal relationships (Ventre Lespiaucq *et al.* 2021). In this sense, seedlings exhibited two groups of fungal associations depending on their subterranean morphology: *Coprinellus* sp. (Psathyrellaceae) in rhizomes, which were closely related to those isolated of mature *C. appendiculata* in Japan for a symbiotic seed experiment (Yagame *et al.* 2013), and rhizoctonia (Ceratobasidiaceae and Serendipitaceae) in the seedling roots. Analogously to mature *C. appendiculata* from different populations (Yagame *et al.* 2021), we can confirm a link between subterranean root system morphology and the type of fungal partners in seedlings.

Overall, ^15^N enrichments of seedlings and protocorms were alike, implying the presence of some fungal derived organic matter in *C. appendiculata* seedlings. Investigated seedlings possessed an intermediate position in ^13^C relative abundances between protocorms and adults, but with a considerable variation. We, therefore, suppose the seedling stage of *C. appendiculata* to be a transition phase between the protocorm stage and adulthood regarding nutrition with increasing independence on fungal derived carbon.

The degree of mycoheterotrophic nutrition in green orchids is apparently modulated by the morphology of subterranean organs, like in the case of *Calypso bulbosa* mycorrhizal with wood-decaying fungi (Suetsugu and Matsubayashi 2021a). Further, different types of fungal partners enable a more or less pronounced carbon flux from fungus to orchid reflected by different ^13^C enrichments (Martos *et al.* 2009; Stöckel *et al.* 2014; Schweiger *et al.* 2018). Thus, the large variance in the ^13^C signature of seedlings could be explained by the combination of rhizoctonia in roots and *Coprinellus* sp. in rhizomes. Depending on the seedling’s age and its need for fungal carbon supply, either rhizoctonia or *Coprinellus* sp. may be more critical fungal partners and be mirrored in the stable isotope signature. While *Coprinellus* sp. mycobionts may enable a distinct partially mycoheterotrophic nutrition of younger seedlings, more developed seedlings might have already become detached from rhizomes and switched to rhizoctonia fungal partners being less or even independent on fungal carbon. In future, this tendency could be reinforced by additional seedling samples at different ages and ^2^H analysis.

### Adults

Assessing three different ontogenetic development stages of one *C. appendiculata* population, we found a step-by-step mycobiont turnover from Psathyrellaceae at protocorm stage to rhizoctonia fungi because Serendipitaceae were dominant mycobionts in roots of adults. Investigated *C. appendiculata* did not feature coralloid rhizomes; thus, the overall change in mycobionts was concomitant with a change in subterranean morphology during the ontogenetic development of studied *C. appendiculata.* Similar ontogenetic changes in fungal community have been reported before, e.g. for *Tipularia discolour* with wood-decomposing Auriculariales as mycobiont of protocorms and Tulasnella as fungal partners at adulthood (McCormick *et al.* 2004). Among achlorophyllous orchid *Gastrodia elata*, *Mycena* were identified in protocorms, while *Armillaria* were detected in tubers of adults (Park and Lee 2013; Chen *et al.* 2019).

Yet, investigated mature *C. appendiculata* individuals were truly autotrophic and had no additional fungal N source, as they did not differ from autotrophic reference plants in enrichment factors ε^13^C, ε^2^H and ε^15^N, and leaf N concentration. In this respect, *C. appendiculata* adults stand out against the majority of the so far investigated orchids with rhizoctonia fungal partners that showed distinct enrichment, particularly in ^2^H (Table 2). Because fungus-to-orchid organic carbon transfer is—to current knowledge—a relatively widespread phenomenon among terrestrial chlorophyllous orchids (Schweiger *et al.* 2019; Gebauer and Clemens 2021), *C. appendiculata* belongs thereby to a minority of orchids that can be truly autotrophic as a mature plant.

However, our results contrast to findings on *C. appendiculata* var. *variabilis* individuals from Japan, which showed the same subterranean morphology and type of mycobiont but have been proposed as ‘cryptic’ mycoheterotrophic (*sensu*Hynson *et al.* 2013) due to enrichment in ^15^N relative to autotrophic reference plants (Yagame *et al.* 2021). Yet, mature *C. appendiculata* can be partially mycoheterotrophic when having already accomplished the switch from rhizoctonia fungal partners to saprotrophic non-rhizoctonia Psathyrellaceae fungi and forming coralloid rhizomes (Suetsugu *et al.* 2021a; Yagame *et al.* 2021), traits similar to fully mycoheterotrophic *C. aphylla* (Yagame *et al.* 2018; Suetsugu *et al.* 2021a). Therefore, mature *C. appendiculata*, hitherto referred to as a partially mycoheterotrophic species during adulthood, has been attributed high flexibility regarding adaption to diverse environmental conditions because it can gain carbon from various fungal partners (Yagame *et al.* 2021). Providing evidence that *C. appendiculata* is capable of a genuinely autotrophic nutrition during adulthood extends our knowledge of *C. appendiculata*’s plasticity by adding another important puzzle piece.

## Conclusions

Though usually found in light-limited forests grounds, *C. appendiculata* develops relatively large green leaves. We provide novel, explicit evidence that mature *C. appendiculata* individuals with rhizoctonia mycobionts can be entirely autotrophic. *Cremastra appendiculata* is, therefore, a notable species among the Orchidaceae as it incorporates the entire spectrum from mycoheterotrophy to autotrophy.

Further, this is the first study to demonstrate dramatic, rather gradual than sudden changes in nutritional mode, root morphology and mycobionts throughout the development of *C. appendiculata* considering the transitional seedling stage. This illustrates how dynamic these aspects can be within orchid individuals.

Besides high within-species variability in fungal partners and nutrition of *C. appendiculata* depending on environmental conditions (Yagame *et al.* 2021), we highlight the importance of developmentally driven changes in mycobionts according to the orchid’s physiological needs, notably ensuring carbon supply with fungal support or independently. Therefore, we suggest high within-individual flexibility of *C. appendiculata* regarding its mycobionts and carbon acquisition, which challenges our current, relatively static view of orchid–fungi interactions on species level.

Illuminating under which spatial and temporal environmental conditions *C. appendiculata* features which nutrition mode could help to deepen our understanding of the mechanisms that contributed to the evolution of saprotroph–mycoheterotrophic plant–fungus interactions.

## Supporting Information

The following additional information is available in the online version of this article—

**Table S1.** Equipment and conditions as used for stable isotope abundance analysis.

**Table S2.** Summary of fungal operational taxonomic units (OTUs)^a^ and their frequencies^b^ detected in protocorms, seedling rhizomes, seedling roots and adult roots of *Cremastra appendiculata* using the Illumina Miseq platform. ^a^OTUs were defined at 3 % sequence dissimilarity using the UPARSE pipeline described in Edgar (2013). Only OTUs representing fungal taxa were retained during analysis, and only those OTUs with >100 total sequences are included here. ^b^Normalized OTU frequencies are indicated by total number of sequences obtained and the percentage of total sequences that each OTU was detected upon.

**Table S3.** Mean and single δ^15^N, δ^13^C, δ^2^H, δ^18^O values, enrichment factors ε^15^N, ε^13^C, ε^2^H, ε^18^O and total nitrogen concentration data of *Cremastra appendiculata* adults, protocorms and seedlings from Fagaceae forest site at Mei Feng, Nantou County, Taiwan (24°05ʹ01.4″N, 121°10ʹ10.2″E, 2000 m a.s.l.).

**Table S4.** Mean and single δ^15^N, δ^13^C, δ^2^H, δ^18^O values, enrichment factors ε^15^N, ε^13^C, ε^2^H, ε^18^O and total nitrogen concentration data of autotrophic reference species from Fagaceae forest site at Mei Feng, Nantou County, Taiwan (24°05ʹ01.4″N, 121°10ʹ10.2″E, 2000 m a.s.l.).

**Figure S1.** Operational taxonomic unit (OTU) rarefaction curves of protocorms, seedling rhizomes, seedling roots and adult roots samples by randomly selecting smaller fractions of reads 100 times.

**Figure S2.** The Bayesian tree based on the sequences of internal transcribed spacer (ITS) nuclear ribosomal DNA of Psathyrellaceae fungi (OL449677) obtained from protocorms and seedling rhizomes of *Cremastra appendiculata* and GenBank database (Yagame *et al.* 2013; Suetsugu *et al.* 2021a). The values above branches are Bayesian posterior probabilities (>70 %).

**Figure S3.** The Bayesian tree based on the sequences of internal transcribed spacer (ITS) nuclear ribosomal DNA of Sebacinales fungi (OL449680) obtained from seedling roots and adult roots of *Cremastra appendiculata* and GenBank database (Yagame *et al.* 2016). The values above branches are Bayesian posterior probabilities (>70 %).

**Figure S4.** The Bayesian tree based on the sequences of internal transcribed spacer (ITS) nuclear ribosomal DNA of Ceratobasidiaceae fungi (OL449678 and OL449679) obtained from seedling roots and adult roots of *Cremastra appendiculata* and GenBank database (Suetsugu *et al.* 2020). The values above branches are Bayesian posterior probabilities (>70 %).

plac021_suppl_Supplementary_Materials

plac021_suppl_Supplementary_Table_S1

## Data Availability

For all metagenome data of this investigation, the accession on NCBI Sequence Read Archive is: PRJNA824353. For all single isotope abundance data, **see****Supporting Information—Tables S3****and****S4**.

## Notes

**Natural History and Conservation. Chief Editor**: F. Xavier Picó
