# Supplementary material for: Fungal association and root morphology shift stepwise during ontogenesis of orchid Cremastra appendiculata towards autotrophic nutrition
Source: AoB Plants. 2022 May 9;14(3):plac021. doi: 10.1093/aobpla/plac021 (PMC9167560; doi:10.1093/aobpla/plac021)
Supplement: plac021_suppl_Supplementary_Materials [file plac021_suppl_supplementary_materials.docx]

**AoB PLANTS Supplementary data**

**Article title: Fungal association and root morphology shift stepwise during ontogenesis of orchid *Cremastra appendiculata* towards autotrophic nutrition**

Table S2. Summary of fungal operational taxonomic units (OTUs)^a^ and their frequencies^b^ detected in protocorms, seedling rhizomes, seedling roots and adult roots of *Cremastra appendiculata* using the Illumina Miseq platform. ^a^OTUs were defined at 3% sequence dissimilarity using the UPARSE pipeline described in Edgar (2013). Only OTUs representing fungal taxa were retained during analysis, and only those OTUs with >200 total sequences are included here. ^b^Normalized OTU frequencies are indicated by total number of sequences obtained and the percentage of total sequences that each OTU was detected upon.

| **OTU ID** | **Protocorms** | | **Seeding rhizomes** | | **Seedling roots** | | **Adult roots** | | **BLAST Identity** | **Closer GenBank Match** | |  |
| --- | --- | --- | --- | --- | --- | --- | --- | --- | --- | --- | --- | --- |
|  | **Sequences** | **Percent** | **Sequences** | **Percent** | **Sequences** | **Percent** | **Sequences** | **Percent** |  | **Accession** | **Length (bp)** | **Match (%)** |
| **Otu1** | **31108** | **75.7** | **23767** | **57.86** | **13** | **0.03** | **28** | **0.07** | **Coprinellus sp. MH-2019a** | **MN622747** | **199** | **99** |
| **Otu2** | **285** | **0.69** | **188** | **0.46** | **9708** | **23.68** | **1439** | **3.51** | **Ceratobasidiaceae sp. CBS 605.93** | **KF267012** | **199** | **99** |
| Otu3 | 574 | 1.4 | 2 | 0.005 | 0 | 0 | 15735 | 38.26 | **Dactylonectria sp.** | **MK336502** | **198** | **99** |
| **Otu4** | **370** | **0.9** | **914** | **2.23** | **2039** | **4.97** | **7592** | **18.46** | **Uncultured Sebacinales clone 6369K3** | **FJ556807** | **188** | **93** |
| Otu5 | 4941 | 12.02 | 0 | 0 | 93 | 0.23 | 4062 | 9.88 | **Uncultured fungus clone Otu397** | **MK983820** | **200** | **100** |
| **Otu6** | **0** | **0** | **2** | **0.005** | **3306** | **8.06** | **1360** | **3.31** | **Rhizoctoniasolani isolate DB336** | **MT950077** | **180** | **89** |
| Otu7 | 0 | 0 | 10090 | 24.56 | 0 | 0 | 0 | 0 | **Uncultured soil fungus clone 30-5** | **DQ421072** | **198** | **99** |
| Otu8 | 27 | 0.07 | 0 | 0 | 463 | 1.13 | 1582 | 3.85 | **Ilyonectria sp. JCM 28414** | **LC133847** | **200** | **100** |
| Otu9 | 2 | 0.005 | 0 | 0 | 1484 | 3.62 | 433 | 1.05 | **Uncultured fungus clone HIB44** | **JX457077** | **184** | **88** |
| Otu10 | 1105 | 2.7 | 0 | 0 | 1825 | 4.45 | 166 | 0.4 | **Cyphellophora sp. isolate** | **MT453284** | **200** | **100** |
| Otu11 | 434 | 1.06 | 0 | 0 | 4243 | 10.33 | 555 | 1.35 | **Nectriaceae sp.** | **KT182907** | **200** | **100** |
| Otu12 | 0 | 0 | 1340 | 3.26 | 0 | 0 | 0 | 0 | **Uncultured Cladophialophora clone** | **KT728346** | **200** | **100** |
| Otu13 | 0 | 0 | 1337 | 3.25 | 0 | 0 | 0 | 0 | **Fungal sp. isolate OTU_410_172** | **MH624468** | **199** | **99** |
| Otu14 | 0 | 0 | 0 | 0 | 0 | 0 | 931 | 2.26 | **Uncultured fungus clone OTU210** | **MN835382** | **196** | **97** |
| Otu15 | 0 | 0 | 0 | 0 | 114 | 0.28 | 793 | 1.93 | **Uncultured Helotiales clone FMr18** | **KT768252** | **189** | **94** |
| Otu16 | 0 | 0 | 1922 | 4.68 | 0 | 0 | 0 | 0 | **uncultured Cantharellales** | **LS977708** | **153** | **99** |
| Otu17 | 40 | 0.1 | 0 | 0 | 608 | 1.48 | 86 | 0.21 | **Uncultured fungus clone OTU505** | **MN835787** | **188** | **94** |
| Otu18 | 5 | 0.01 | 0 | 0 | 678 | 1.65 | 114 | 0.28 | **Uncultured soil fungus clone BD99** | **JQ666722** | **196** | **98** |
| Otu19 | 0 | 0 | 0 | 0 | 1 | 0.002 | 611 | 1.49 | **Uncultured soil fungus clone SK39** | **JQ666560** | **197** | **97** |
| Otu20 | 10 | 0.02 | 0 | 0 | 779 | 1.91 | 100 | 0.24 | **Volutella ciliata strain HSP113** | **MK433634** | **199** | **99** |
| Otu21 | 19 | 0.05 | 0 | 0 | 772 | 1.88 | 156 | 0.38 | **Plectosphaerellacucumerina clone SF_25** | **MT529301** | **200** | **100** |
| Otu22 | 4 | 0.01 | 0 | 0 | 690 | 1.68 | 48 | 0.12 | **Uncultured Basidiomycota clone** | **GU328503** | **186** | **92** |

Table S2 (continued)

| Otu23 | 0 | 0 | 0 | 0 | 223 | 0.54 | 348 | 0.85 | **Beauveria pseudobassiana isolate C5** | **MK142275** | **200** | **100** |
| --- | --- | --- | --- | --- | --- | --- | --- | --- | --- | --- | --- | --- |
| Otu24 | 645 | 1.57 | 0 | 0 | 1 | 0.002 | 0 | 0 | **Paecilomycesnostocoides isolate** | **MT924812** | **171** | **90** |
| Otu25 | 0 | 0 | 0 | 0 | 461 | 1.12 | 86 | 0.21 | **Orbiliaceae sp. clone OTU1774** | **MK537232** | **167** | **83** |
| Otu26 | 6 | 0.01 | 0 | 0 | 505 | 1.23 | 143 | 0.35 | **Paecilomyces sp. strain MR94** | **KY031682** | **200** | **100** |
| Otu27 | 54 | 0.13 | 0 | 0 | 550 | 1.34 | 22 | 0.05 | **Densocarpashanorii voucher RH136** | **KT361850** | **160** | **97** |
| Otu28 | 0 | 0 | 4 | 0.01 | 564 | 1.38 | 0 | 0 | **Fusarium sp. strain GFR28** | **MT447533** | **200** | **100** |
| Otu29 | 3 | 0.03 | 0 | 0 | 643 | 1.57 | 179 | 0.44 | **Tylosporafibrillosa isolate 160** | **MN047014** | **60** | **95** |
| Otu30 | 4 | 0.01 | 0 | 0 | 311 | 0.76 | 158 | 0.38 | **Uncultured fungus isolate OTU0691** | **MT925017** | **172** | **82** |
| Otu32 | 0 | 0 | 0 | 0 | 721 | 1.76 | 27 | 0.07 | **Tetracladium sp.** | **MZ493007** | **198** | **99** |
| Otu34 | 12 | 0.03 | 0 | 0 | 314 | 0.77 | 0 | 0 | **Uncultured Helotiales genomic DNA** | **HG936491** | **195** | **98** |
| Otu35 | 2 | 0.005 | 0 | 0 | 346 | 0.84 | 9 | 0.02 | **Pochoniasuchlasporia genes** | **AB214658** | **200** | **100** |
| Otu36 | 12 | 0.03 | 0 | 0 | 305 | 0.74 | 15 | 0.04 | **Herpotrichiellaceae sp.** | **KM816771** | **151** | **98** |
| Otu37 | 0 | 0 | 0 | 0 | 0 | 0 | 265 | 0.64 | **Uncultured fungus isolate OTU_229** | **MH238730** | **164** | **90** |
| Otu38 | 0 | 0 | 0 | 0 | 99 | 0.24 | 194 | 0.47 | **Uncultured fungus clone S2_31639** | **KU000477** | **175** | **96** |
| Otu39 | 0 | 0 | 0 | 0 | 267 | 0.65 | 26 | 0.06 | **Fungal sp. isolate SF06** | **MN727895** | **200** | **100** |
| Otu40 | 44 | 0.11 | 0 | 0 | 242 | 0.59 | 0 | 0 | **Uncultured fungus isolate KTRF569** | **MG433496** | **183** | **92** |
| Otu41 | 0 | 0 | 0 | 0 | 336 | 0.82 | 59 | 0.14 | **Uncultured fungus clone G01** | **JF433010** | **185** | **89** |
| Otu42 | 0 | 0 | 0 | 0 | 0 | 0 | 254 | 0.62 | **Uncultured fungus clone Otu50** | **MK983902** | **200** | **100** |
| Otu44 | 0 | 0 | 271 | 0.66 | 0 | 0 | 0 | 0 | **Pochoniachlamydosporia** | **AB709852** | **200** | **100** |
| Otu45 | 14 | 0.03 | 0 | 0 | 169 | 0.41 | 22 | 0.05 | **Fusarium sp.** | **MK808263** | **181** | **88** |
| Otu46 | 0 | 0 | 0 | 0 | 156 | 0.38 | 22 | 0.05 | **Uncultured fungus clone** | **KC965937** | **194** | **97** |
| Otu47 | 0 | 0 | 0 | 0 | 188 | 0.46 | 0 | 0 | **Odontia sp. isolate ongi2** | **MG719980** | **191** | **96** |
| Otu50 | 0 | 0 | 219 | 0.53 | 0 | 0 | 0 | 0 | **Cladophialophora sp. TS-2016** | **KT777711** | **200** | **100** |
| Otu54 | 0 | 0 | 0 | 0 | 186 | 0.45 | 5 | 0.01 | **Bloxamia sp. TNS:F24589** | **LC169493** | **200** | **100** |
| Otu59 | 13 | 0.03 | 0 | 0 | 255 | 0.62 | 0 | 0 | **Ganoderma sp. isolate DWS(19)-46** | **MK605943** | **200** | **100** |
| Otu65 | 265 | 0.64 | 0 | 0 | 0 | 0 | 0 | 0 | **Uncultured fungus clone Otu439** | **MK983853** | **200** | **100** |
| Otu67 | 0 | 0 | 369 | 0.9 | 0 | 0 | 0 | 0 | **Lecanicilliumpsalliotae** | **LR778252** | **200** | **100** |
| Otu75 | 0 | 0 | 0 | 0 | 156 | 0.38 | 65 | 0.16 | **Fungal sp. isolate F_OTU_551** | **MT353534** | **71** | **93** |

Table S3: Mean and single δ^15^N, δ^13^C, δ^2^H, δ^18^O values, enrichment factors ε^15^N, ε^13^C, ε^2^H, ε^18^O and total nitrogen concentration data of *Cremastra appendiculata* adults, protocorms and seedlings from Fagaceae forest site at Mei Feng, Nantou County, Taiwan (24°05'01.4"N 121°10'10.2"E, 2000 m. a. s. l.).

| **Species, Sample type** |  | **Plot** | **Total N [mmol g_dw_^-1^]** | **δ^15^N (‰)** | **δ^13^C (‰)** | **δ^2^H (‰)** | **δ^18^O (‰)** | **ε^15^N (‰)** | **ɛ^13^C (‰)** | **ɛ^2^H (‰)** | **ɛ^18^O (‰)** |
| --- | --- | --- | --- | --- | --- | --- | --- | --- | --- | --- | --- |
| *Cremastra appendiculata* (D.Don) Makino, |  | **Mean** | **2.52** | **-3.85** | **-32.82** | **-82.99** | **18.09** | **0.46** | **0.84** | **1.80** | **-1.14** |
| **Adult** |  | ***SD*** | ***0.18*** | ***0.67*** | ***0.59*** | ***6.30*** | ***0.46*** | ***0.99*** | ***0.72*** | ***4.59*** | ***0.58*** |
|  |  | 2011-1 | 2.69 | -3.88 | -32.02 | -80.61 | 17.34 | 0.40 | 1.97 | -0.19 | -1.89 |
|  |  | 2011-2 | 2.31 | -4.20 | -33.34 | -85.36 | 18.18 | 0.55 | 0.94 | 1.24 | -0.79 |
|  |  | 2011-3 | 2.55 | -4.30 | -32.65 | -92.36 | 18.27 | -0.85 | 0.07 | -4.40 | -1.25 |
|  |  | 2011-4 | 2.69 | -2.68 | -32.62 | -81.16 | 18.07 | 1.92 | 0.82 | 4.86 | -1.38 |
|  |  | 2011-5 | 2.35 | -4.17 | -33.46 | -75.48 | 18.57 | 0.25 | 0.41 | 7.49 | -0.38 |
|  |  |  |  |  |  |  |  |  |  |  |  |
| *Cremastra appendiculata* (D.Don) Makino, |  | **Mean** | **3.26** | **0.33** | **-25.41** | **-55.97** | **15.68** | **2.98** | **8.00** | **28.83** | **-3.55** |
| **Protocorm** |  | ***SD*** | ***0.28*** | ***2.33*** | ***0.46*** | ***6.54*** | ***1.79*** | ***1.36*** | ***0.60*** | ***5.80*** | ***1.56*** |
|  |  | 2011-1 | 3.36 | -2.32 | -25.60 | -47.12 | 16.99 | 1.97 | 8.39 | 33.30 | -2.24 |
|  |  | 2011-2 | 3.59 | 0.23 | -25.56 | -60.48 | 13.46 | 4.98 | 8.73 | 26.12 | -5.51 |
|  |  | 2011-3 | 2.93 | -2.35 | -25.98 | -58.44 | 17.14 | 1.10 | 6.74 | 29.52 | -2.38 |
|  |  | 2011-4 | 3.65 | -1.84 | -25.29 | -51.22 | 16.80 | 2.77 | 8.15 | 34.80 | -2.65 |
|  |  | 2011-5 | 3.16 | 0.58 | -25.83 | -62.58 | 13.99 | 4.99 | 8.04 | 20.39 | -4.96 |
|  |  |  |  |  |  |  |  |  |  |  |  |
|  |  | 2015-1 | 3.39 | 3.11 | -24.81 | NA | NA | 2.83 | 8.14 | NA | NA |
|  |  | 2015-2 | 2.91 | 3.20 | -24.66 | NA | NA | 2.63 | 8.21 | NA | NA |
|  |  | 2015-3 | 3.07 | 2.05 | -25.51 | NA | NA | 2.59 | 7.60 | NA | NA |
|  |  |  |  |  |  |  |  |  |  |  |  |
| *Cremastra appendiculata* (D.Don) Makino, |  | **Mean** | **2.76** | **4.26** | **-29.92** | NA | NA | **4.15** | **3.06** | NA | NA |
| **Seedling** |  | ***SD*** | ***1.03*** | ***1.23*** | ***3.51*** | NA | NA | ***1.57*** | ***3.59*** | NA | NA |
|  |  | 2015-1 | 3.64 | 5.31 | -27.22 | NA | NA | 5.03 | 5.74 | NA | NA |
|  |  | 2015-2 | 1.63 | 2.91 | -33.89 | NA | NA | 2.34 | -1.02 | NA | NA |
|  |  | 2015-3 | 3.00 | 4.55 | -28.65 | NA | NA | 5.09 | 4.45 | NA | NA |
|  |  |  |  |  |  |  |  |  |  |  |  |

Table S4: Mean and single δ^15^N, δ^13^C, δ^2^H, δ^18^O values, enrichment factors ε^15^N, ε^13^C, ε^2^H, ε^18^O and total nitrogen concentration data of autotrophic reference species from Fagaceae forest site at Mei Feng, Nantou County, Taiwan (24°05'01.4"N 121°10'10.2"E, 2000 m. a. s. l.). Nomenclature follows the International Plant Names Index (IPNI, 2021).

| **Species (Family)** |  | **Plot** | **Total N [mmol g_dw_^-1^]** | **δ^15^N (‰)** | **δ^13^C (‰)** | **δ^2^H (‰)** | **δ^18^O (‰)** | **ε^15^N (‰)** | **ɛ^13^C (‰)** | **ɛ^2^H (‰)** | **ɛ^18^O (‰)** |
| --- | --- | --- | --- | --- | --- | --- | --- | --- | --- | --- | --- |
| **Autotrophic reference plants** |  | **Mean** | **2.53** | **-2.87** | **-33.44** | **-84.79** | **19.22** | **0.00** | **0.00** | **0.00** | **0.00** |
|  |  | ***SD*** | ***0.49*** | ***2.53*** | ***1.27*** | ***8.20*** | ***2.34*** | ***0.48*** | ***0.32*** | ***2.78*** | ***0.73*** |
|  |  |  |  |  |  |  |  |  |  |  |  |
| *Selaginella delicatula* (Desv. ex Poir.) Alston |  | 2011-1 | 2.26 | -5.50 | -33.22 | -71.77 | 19.69 | -1.22 | 0.78 | 8.65 | 0.46 |
| (Selaginellaceae) |  | 2011-2 | 2.72 | -3.37 | -33.49 | -89.11 | 14.69 | 1.39 | 0.80 | -2.51 | -4.28 |
|  |  | 2011-3 | 2.96 | -5.01 | -32.69 | -91.99 | 20.86 | -1.56 | 0.03 | -4.03 | 1.34 |
|  |  | 2011-4 | 2.19 | -6.96 | -31.66 | -90.21 | 20.28 | -2.35 | 1.78 | -4.19 | 0.83 |
|  |  | 2011-5 | 3.00 | -5.91 | -32.62 | -91.26 | 18.32 | -1.49 | 1.25 | -8.29 | -0.63 |
|  |  |  |  |  |  |  |  |  |  |  |  |
| *Oreocnide pedunculata* (Shirai) Masam. |  | 2011-1 | 2.53 | -4.26 | -33.31 | -82.66 | 15.54 | 0.02 | 0.69 | -2.24 | -3.69 |
| (Urticaceae) |  | 2011-2 | 3.03 | -5.08 | -33.08 | -79.40 | 20.19 | -0.32 | 1.20 | 7.20 | 1.22 |
|  |  | 2011-3 | 2.49 | -4.69 | -33.18 | -90.44 | 15.30 | -1.23 | -0.47 | -2.48 | -4.22 |
|  |  | 2011-4 | 2.90 | -2.83 | -32.91 | -77.45 | 15.95 | 1.78 | 0.53 | 8.57 | -3.50 |
|  |  | 2011-5 | 2.64 | -4.38 | -33.08 | -85.74 | 15.83 | 0.04 | 0.79 | -2.77 | -3.12 |
|  |  |  |  |  |  |  |  |  |  |  |  |
| *Begonia palmata* D.Don |  | 2011-1 | 2.69 | -1.88 | -34.24 | -86.19 | 21.74 | 2.41 | -0.25 | -5.77 | 2.51 |
| (Begoniaceae) |  | 2011-2 | 2.42 | -6.19 | -36.09 | -102.12 | 20.90 | -1.44 | -1.81 | -15.52 | 1.93 |
|  |  | 2011-3 | 2.48 | -3.67 | -32.24 | -92.16 | 21.89 | -0.22 | 0.47 | -4.20 | 2.37 |
|  |  | 2011-4 | 2.79 | -5.17 | -33.94 | -85.36 | 23.65 | -0.57 | -0.49 | 0.66 | 4.20 |
|  |  | 2011-5 | 2.88 | -3.73 | -34.49 | -84.23 | 22.35 | 0.69 | -0.62 | -1.26 | 3.40 |
|  |  |  |  |  |  |  |  |  |  |  |  |

Table S4 (continued)

| **Species (Family)** |  |  | **Plot** | **Total N [mmol g_dw_^-1^]** | **δ^15^N (‰)** | **δ^13^C (‰)** | **δ^2^H (‰)** | **δ^18^O (‰)** | **ε^15^N (‰)** | **ɛ^13^C (‰)** | **ɛ^2^H (‰)** | **ɛ^18^O (‰)** |
| --- | --- | --- | --- | --- | --- | --- | --- | --- | --- | --- | --- | --- |
| *Oplismenus compositus* (L.) P.Beauv. |  |  | 2011-1 | 2.67 | -4.43 | -35.37 | -91.49 | 18.25 | -0.14 | -1.38 | -11.07 | -0.98 |
| (Poaceae) |  |  | 2011-2 | 2.54 | -5.25 | -35.23 | -88.51 | 18.54 | -0.49 | -0.95 | -1.91 | -0.43 |
|  |  |  | 2011-3 | 2.86 | -3.62 | -34.42 | -87.57 | 19.61 | -0.16 | -1.70 | 0.39 | 0.09 |
|  |  |  | 2011-4 | 2.93 | -4.88 | -33.84 | -93.29 | 18.92 | -0.28 | -0.39 | -7.27 | -0.53 |
|  |  |  | 2011-5 | 2.91 | -4.30 | -36.08 | -85.99 | 19.77 | 0.12 | -2.21 | -3.02 | 0.82 |
|  |  |  |  |  |  |  |  |  |  |  |  |  |
| *Alpinia sessiliflora* Kitam. (Zingiberaceae) |  |  | 2011-1 | 1.48 | -5.36 | -33.83 | -70.01 | 20.93 | -1.07 | 0.16 | 10.41 | 1.70 |
| (Zingiberaceae) |  |  | 2011-2 | 1.71 | -3.90 | -33.53 | -73.85 | 20.50 | 0.85 | 0.76 | 12.75 | 1.53 |
|  |  |  | 2011-3 | 1.60 | -0.27 | -31.05 | -77.63 | 19.96 | 3.18 | 1.67 | 10.33 | 0.44 |
|  |  |  | 2011-4 | 1.69 | -3.19 | -34.87 | -83.78 | 18.44 | 1.42 | -1.42 | 2.24 | -1.01 |
|  |  |  | 2011-5 | 1.48 | -3.78 | -33.08 | -67.67 | 18.46 | 0.64 | 0.79 | 15.30 | -0.49 |
|  |  |  |  |  |  |  |  |  |  |  |  |  |
| [*Gynostemma pentaphyllum* (Thunb.) Makino](http://www.theplantlist.org/tpl1.1/record/kew-2836473) |  |  | 2015-1 | 2.35 | -0.22 | -33.34 | NA | NA | -0.50 | -0.38 | NA | NA |
| (Cucurbitaceae) |  |  | 2015-2 | 2.14 | 0.38 | -33.25 | NA | NA | -0.19 | -0.38 | NA | NA |
|  |  |  | 2015-3 | 2.66 | -2.80 | -34.81 | NA | NA | -2.25 | -1.70 | NA | NA |
|  |  |  |  |  |  |  |  |  |  |  |  |  |
| *Pilea melastomoides* (Poir.) Wedd. |  |  | 2015-1 | 3.44 | -1.47 | -34.79 | NA | NA | -1.75 | -1.84 | NA | NA |
| (Urticaceae) |  |  | 2015-2 | 2.98 | -0.17 | -34.22 | NA | NA | -0.73 | -1.35 | NA | NA |
|  |  |  | 2015-3 | 3.12 | -0.82 | -33.90 | NA | NA | -0.27 | -0.80 | NA | NA |
|  |  |  |  |  |  |  |  |  |  |  |  |  |
| *Eleutherococcus trifoliatus* (L.) S.Y.Hu |  |  | 2015-1 | 2.26 | 2.24 | -32.23 | NA | NA | 1.96 | 0.73 | NA | NA |
| (Araliaceae) |  |  | 2015-2 | 2.04 | 2.25 | -32.22 | NA | NA | 1.68 | 0.65 | NA | NA |
|  |  |  | 2015-3 | 2.00 | 2.03 | -31.89 | NA | NA | 2.57 | 1.22 | NA | NA |
|  |  |  |  |  |  |  |  |  |  |  |  |  |
| *Onychium contiguum* |  |  | 2015-1 | 2.83 | 0.58 | -31.46 | NA | NA | 0.30 | 1.49 | NA | NA |
| (Adiantaceae) |  |  | 2015-2 | 3.00 | -0.19 | -31.79 | NA | NA | -0.76 | 1.08 | NA | NA |
|  |  |  | 2015-3 | 2.84 | -0.58 | -31.81 | NA | NA | -0.04 | 1.29 | NA | NA |
|  |  |  |  |  |  |  |  |  |  |  |  |  |


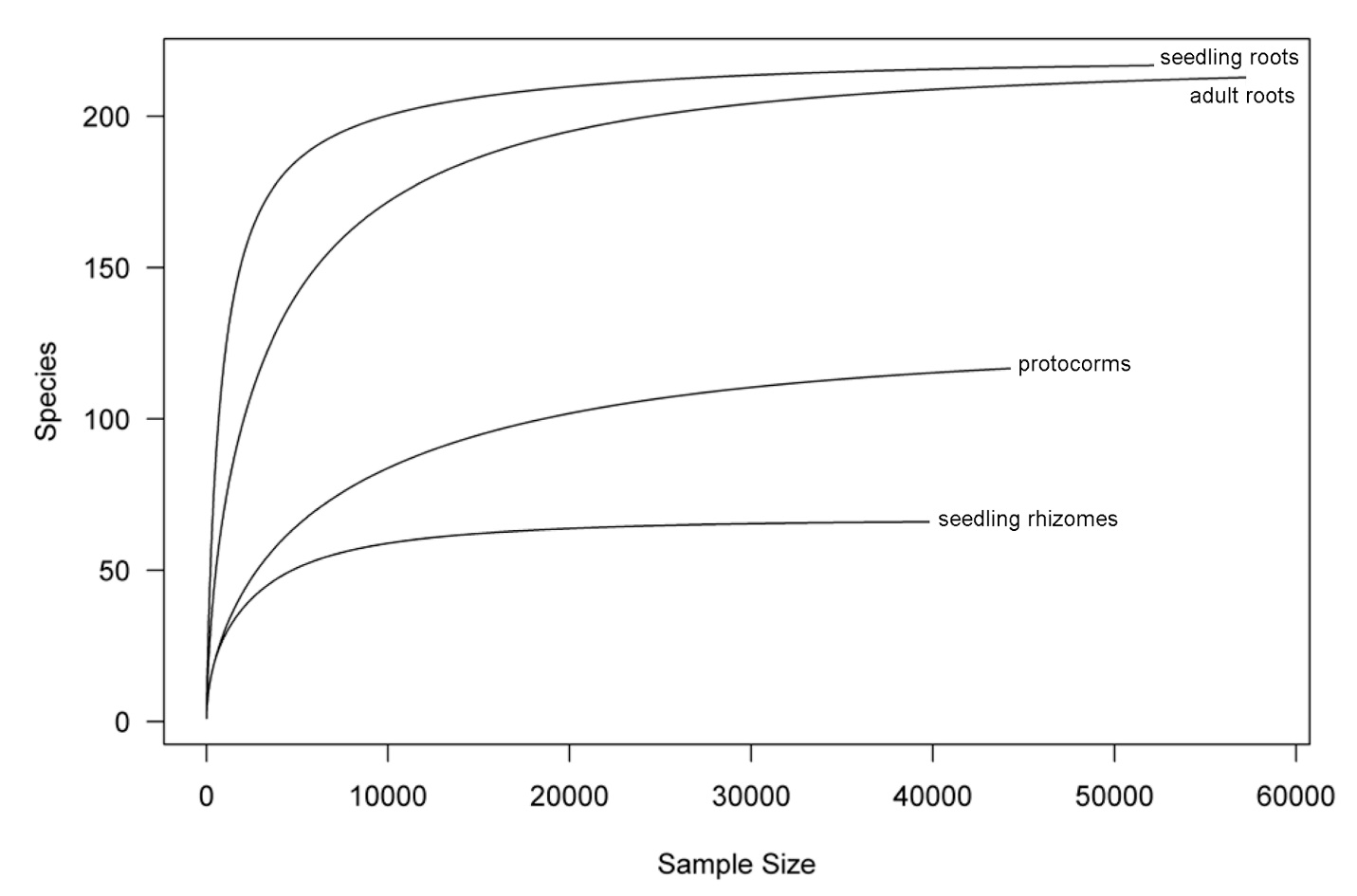


Fig. S1. OTU rarefaction curves of protocorms, seedling rhizomes, seedling roots and adult roots samples by randomly selecting smaller fractions of reads 100 times.





Fig. S2. The Bayesian tree based on the sequences of internal transcribed spacer (ITS) nuclear ribosomal DNA of Psathyrellaceae fungi (OL449677) obtained from protocorms and seedling rhizomes of *Cremastra appendiculata* and GenBank database (Yagame *et al.*, 2013; Suetsugu *et al.*, 2021c). The values above branches are Bayesian posterior probabilities (> 70 %).





Fig. S3. The Bayesian tree based on the sequences of internal transcribed spacer (ITS) nuclear ribosomal DNA of Sebacinales fungi (OL449680) obtained from seedling roots and adult roots of *Cremastra appendiculata* and GenBank database (Yagame *et al.*, 2016). The values above branches are Bayesian posterior probabilities (> 70%).





Fig. S4. The Bayesian tree based on the sequences of internal transcribed spacer (ITS) nuclear ribosomal DNA of Ceratobasidiaceae fungi (OL449678 and OL449679) obtained from seedling roots and adult roots of *Cremastra appendiculata* and GenBank database (Suetsugu *et al.*, 2020). The values above branches are Bayesian posterior probabilities (> 70%).
