# Supplementary material for: Fungal association and root morphology shift stepwise during ontogenesis of orchid Cremastra appendiculata towards autotrophic nutrition
Source: AoB Plants. 2022 May 9;14(3):plac021. doi: 10.1093/aobpla/plac021 (PMC9167560; doi:10.1093/aobpla/plac021)
Supplement: plac021_suppl_Supplementary_Table_S1 [file plac021_suppl_supplementary_table_s1.docx]

**AoB PLANTS Supplementary data**

**Article title: Fungal association and root morphology shift stepwise during ontogenesis of orchid *Cremastra appendiculata* towards autotrophic nutrition**

Table S1: Equipment and conditions as used for stable isotope abundance analysis

- Sample drying temperature: 105°C
- Sample grinding equipment: Ball mill (Retsch Schwingmühle MM2, Haan, Germany)
- Micro balances: Sartorius CPA2P & MSE3.6P-000-DM, Göttingen, Germany & Mettler AT21, Gießen, Germany)
- For C and N isotope abundance analysis of protocorm samples from 2019: EA-IRMS coupling (otherwise according to Bidartondo *et al.* (2004))
- Elemental analyser: EA IsoLink CN, Thermo Fisher Scientific, Bremen, Germany
- Continuous flow isotope ratio mass spectrometer: delta V advantage, Thermo Fisher Scientific, Bremen, Germany
- Open-Split Interface: ConFlo IV, Thermo Fisher Scientific, Bremen, Germany
- Plant sample weight: 1-2 mg
- Standard: Acetanilide (0.3-1 mg)
- Capsules material: tin
- For H and O isotope abundance analysis: TC-IRMS coupling
- Pyrolysis oven: HTO, HEKAtech, Wegberg, Germany
- Isotope ratio mass spectrometer: delta V plus (Thermo Fisher Scientific, Bremen, Germany)
- Open-split Interface: ConFlo IV (Thermo Fisher Scientific, Bremen,
  Germany)
- Plant sample weight: 0.5-1 mg
- Standard: Benzoic acid
- Capsules material: annealed silver

For H isotope abundance, each sample was measured four times in a row, with the first three measures being rejected to avoid memory effect bias of the previous sample. A bias of post-sampling H atom exchange between organically bound hydroxyl groups of the sample with H_2_O in the ambient air (Yakir, 1992) was minimised by plot-wise analysis of the target orchid samples and their respective reference plant samples in identical batches (Gebauer *et al.*, 2016).

- Substances for calibration of working standards
- CO_2_ vs. V-PDB: IAEA-CH3 and IAEA-CH6
- N_2_ vs. N_2_ in air: N1 and N2
- H_2_ vs. V-SMOW: IAEA-CH7, V-SMOW and SLAP
- CO vs. V-SMOW: IAEA601 and IAEA602

Provider of calibration substances: International Atomic Energy Agency, Vienna, Austria
